# Positional and dimensional temporomandibular joint osseous changes in patients treated with the forsus fatigue resistant device: a non-randomized clinical trial 

**DOI:** 10.1007/s00784-025-06474-3

**Published:** 2025-08-18

**Authors:** Ramy Abdul-Rahman Ishaq, Maged S. Alhammadi, Mona M. Salah Fayed, Sherif A. Elkordy, Najah Alhashimi, Abeer A. Almashraqi

**Affiliations:** 1 Department of Orthodontics, Pedodontics and Preventive Dentistry, Faculty of Dentistry, Sana’a University, Sana’a, Republic of Yemen; 2 Orthodontics and Dentofacial Orthopedics, Department of Preventive Dental Sciences, College of Dentistry, Jazan University, Jazan, Saudi Arabia; 3 Department of Orthodontics, Faculty of Dentistry, Cairo University, Cairo, Egypt; 4 Unit and Divisional Chief Orthodontics at Hamad Medical Corporation, and Associate Professor, College of Dental Medicine, Qatar University, Doha, Qatar; 5 Department of Clinical Oral Health Sciences, College of Dental Medicine, QU Health, Qatar University, Doha, Qatar

**Keywords:** CBCT, Class II malocclusion, Fixed functional appliances, Forsus fatigue resistant device, Temporomandibular joint, Growth modification

## Abstract

**Objective:**

This study aimed to assess the osseous positional and dimensional changes in the temporomandibular joint (TMJ) of patients with skeletal Class II malocclusion treated with the Forsus Fatigue Resistant Device (FFRD).

**Materials and methods:**

This non-randomized clinical trial included 40 female subjects, aged 11 to 15, with skeletal Class II malocclusion. Participants were divided into a treatment and a control group. After alignment and leveling with fixed orthodontic appliances using 0.019 × 0.025-inch stainless-steel archwires, the FFRD was fitted. The overjet was corrected to achieve an edge-to-edge incisor relationship. Cone Beam Computed Tomography (CBCT) images were taken before (T1) and after (T2) the fixed functional phase. The TMJs were assessed for positional and dimensional osseous changes in the mandibular condyles, glenoid fossae, and joint spaces. Intra- and inter-group comparisons were conducted using paired t-tests and independent t-tests, respectively.

**Results:**

The initial measurements of age, cervical stage, anteroposterior and vertical skeletal alignment, and TMJ parameters were similar between the study and control groups. Three participants from the study group were lost to follow-up, resulting in 17 participants completing the trial. In the treatment group, condylar width decreased significantly by 0.52 ± 0.92 mm, in contrast to an increase of 0.17 ± 0.35 mm in the control group (*P* = 0.010). Moreover, the anterior wall inclination in the treatment group was reduced by 3.13 ± 10.77 degrees, compared to an increase of 2.95 ± 4.1 degrees in the control group (*P* = 0.003). All other measurements displayed no significant differences between the two groups.

**Conclusion:**

In the short term, the FFRD redirected the growth of the articular eminence anteriorly, contrasting with the normal growth pattern of untreated individuals. However, no additional positional or dimensional changes in the TMJ were observed.

**Clinical relevance:**

By aligning the jaw and correcting overjet, clinicians can potentially enhance occlusal relationships and contribute to better jaw function. However, it is important to investigate whether this process is associated with any changes in the bony structures of the TMJ. This study underscores the efficacy of the FFRD in reshaping the osseous components of the TMJ, which may lead to improved functional outcomes for patients with skeletal Class II malocclusion.

**Supplementary Information:**

The online version contains supplementary material available at 10.1007/s00784-025-06474-3.

## Introduction

Mandibular deficiency is a common underlying factor in skeletal Class II malocclusion [1]. The optimal treatment for growing patients involves stimulating mandibular growth to achieve functional occlusion and desirable facial profile aesthetics. Several treatment modalities for skeletal Class II malocclusion are available, depending on the underlying cause and the patient’s age at treatment initiation. These include extraoral appliances, removable and fixed functional appliances, camouflage treatments, and surgical procedures [2, 3]. Functional appliances, both removable and fixed, have traditionally been the primary treatment choices for addressing mandibular deficiency during the growth period [4] and remain the recommended approach [5–7].

Fixed functional appliances (FFAs) are compliance-free, tooth-borne devices designed to protrude the mandible forward [1, 8–10]. Popular FFAs such as the Herbst, Jasper Jumper, and Forsus Fatigue Resistant Device (FFRD) are valued for their independence from patient cooperation [11]. The FFRD, in particular, is one of the most widely utilized appliances today [12, 13]. Introduced by William Vogt in 2006 [14], the FFRD’s EZ module is a two-piece, semi-rigid telescoping system. It includes a piston encompassed by a super-elastic nickel-titanium coil spring and can be assembled chair-side [15]. Used in conjunction with fixed orthodontic appliances, the FFRD directly attaches to the molar bands of the upper arch and the main archwire of the lower arch, producing a protrusive force on the lower dental arch [16]. In contrast to the rigid advancement of the Herbst appliance, the FFRD provides flexibility in mandibular positioning [17]. FFRD displaces the mandible forward, translates the condyle out of the condylar fossa, and transmits forces to both the dentition and basal bone [11].

Evidence from animal studies [18–20] and clinical research [21–24] suggests that mandibular growth and condylar development can be stimulated. The hypothesis is that forward positioning of the mandible using FFAs stretches the lateral pterygoid muscles, thus displacing the mandibular condyles within the Glenoid Fossae (GF). This displacement is thought to stimulate remodeling of the anterior fossa and/or the posterior condyle [25]. However, some randomized clinical trials indicated that fixed functional right device (FFRD) use resulted in minimal significant skeletal mandibular changes, with an increase in effective length (Co-Gn) of only 1.8 mm [3, 26, 27]. These findings were later contradicted by subsequent clinical trials, as summarized in systematic reviews [8, 28], which refuted the claim that FFAs induce noteworthy skeletal changes in the mandible. Instead, these reviews found more prominent changes in the dentoalveolar area. Further investigation into the effects of FFAs on the temporomandibular joint (TMJ) might clarify whether the changes involve actual growth and/or remodeling of the condyle and glenoid fossa or are merely positional rather than dimensional changes.

Clinical studies investigating the Herbst appliance have yielded conflicting findings, often due to comparing fixed with removable functional appliances without considering growth influences at this critical age [29–32]. These studies were critically evaluated in a systematic review, which concluded that definitive conclusions could not be drawn [18]. This was due to critical design issues and analytical flaws within the studies. Specifically, the use of two-dimensional cephalometric radiographic imaging has proven inadequate for reliably detecting osseous changes in the TMJ. Consequently, further research utilizing Cone Beam Computed Tomography (CBCT) and including an untreated control group is recommended to eliminate the effects of natural growth [18]. Currently, no clinical study has comprehensively examined the effects of FFRD on the osseous components of the TMJ, underscoring the need for such research. Therefore, this study aimed to evaluate the three-dimensional (3D) positional and dimensional osseous changes in the TMJ due to FFRD in a cohort of skeletal Class II female subjects, compared to a matched untreated control group.

## Materials and methods

### Trial design

This non-randomized clinical trial received ethical approval from the Ethics Committee of the Faculty of Dentistry, Cairo University (approval number IRB: 5/15-07-1220), Cairo, Egypt. All methods adhered to the approved guidelines and regulations. Participants were recruited from those seeking treatment at the outpatient clinic of the Faculty of Dentistry, Cairo University. Candidates were approached during their diagnostic visit and invited to participate in the study. The principal investigator explained the trial to both the children and their parents or guardians. It was clearly communicated that enrollment would involve additional treatment stages and that opting out would not impact the standard treatment they would receive. Written informed consent was obtained from all parents or legal guardians.

### Sample size calculation

The sample size was calculated using G*Power 3.0.10 software (Universität Düsseldorf, Düsseldorf, Germany), with an alpha of 0.05 and a power of 80%, based on a prior study by Arici et al. [33]. That study reported mean changes in the posterior-anterior joint space volume of 34 ± 33 mm^3^ for the control group and 80 ± 49 mm^3^ for the study group. This calculation determined a minimum required sample size of 16 participants per group, which was increased to 20 per group to account for potential dropouts.

### Inclusion and exclusion criteria

The inclusion criteria for the study were: (1) females aged 11 to 15 years; (2) in the permanent dentition stage; (3) at skeletal maturation stage 3–4 of the Cervical Vertebrae Maturational Index (CVMI) [34]; (4) exhibiting a convex facial profile due to mandibular deficiency (SNB ≤ 75°; B-Nv≥−4 mm); (5) possessing a horizontal or average growth pattern (MP/SN ≤ 30°); (6) presenting with Class II Division 1 malocclusion with an overjet ≥ 5 mm and at least a half-unit canine relationship bilaterally; (7) showing minimal crowding in the lower arch (< 5 mm); (8) having proclined or normally inclined upper incisors; and (9) exhibiting retroclined or normally inclined lower incisors.

The exclusion criteria were: (1) clinically diagnosed temporomandibular disorders (TMDs); (2) systemic diseases, syndromic conditions, or craniofacial anomalies; (3) use of chronic medications; (4) history of previous orthopedic or orthodontic treatment; (5) poor oral hygiene and pathological conditions contraindicating orthodontic treatment (such as a high caries index, severe gingivitis, or periodontitis); (6) missing or extracted teeth (excluding third molars); (7) presence of parafunctional habits; and (8) severe proclination of the lower labial segment necessitating extraction in the lower arch. The main investigator (R.A.I) conducted the clinical examination for TMD assessment under the direct supervision of an experienced TMD specialist (M.S.F).

### Intervention

Each participant in the treatment group had 0.022 × 0.028-inch MBT orthodontic brackets (3M Unitek, Monrovia, Calif., USA) attached to both the upper and lower arches. All orthodontic procedures were conducted by a single orthodontist (R.A.I.). A soldered passive transpalatal arch was affixed to the maxillary first molars. The process of alignment and leveling involved engaging 0.019 × 0.025-inch stainless-steel archwires in the upper and lower arches, secured distal to the first molar tubes. The device’s EZ module was installed according to the manufacturer’s instructions provided by 3M Unitek (Monrovia, Calif., USA). The FFRD size was determined using a ruler supplied in the device kit, with measurements taken while the patient was occluding in the Class II position. The distal component of the device was attached to the extraoral tube of the upper molar bands, while the mesial part was crimped onto the archwire between the brackets on the mandibular canines and first premolars. The device’s spring compressed when the patient attempted posterior occlusion, thereby compelling the mandible to protrude forward.

**Fig. 1:**
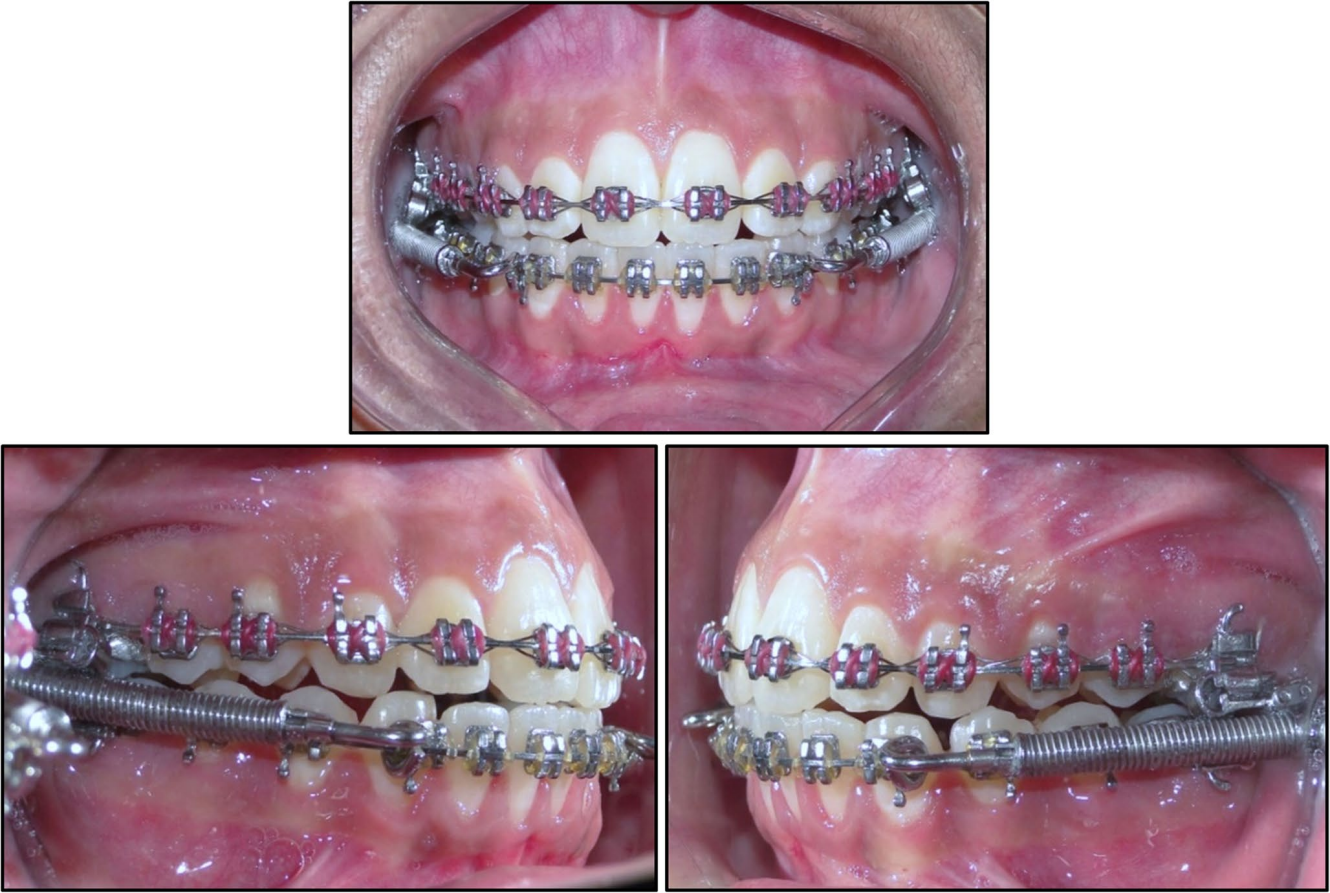
Intraoral photograph demonstrating termination of the fixed functional phase. The incisors are in an edge-to-edge relationship

Instructions on oral hygiene and appliance care were provided. The first follow-up visit was scheduled for 2 weeks after fitting the FFRD to conduct an initial check. Subsequent appointments were arranged at 6-week intervals to monitor treatment progress, as well as to provide maintenance and activation. This involved adding 1 mm stainless-steel crimpable stops to increase spring compression upon closure. The overjet was overcorrected to an edge-to-edge incisal relationship, after which the FFRD was removed (Fig. 1). Treatment continued to finalize the occlusion, with Class II elastics used to maintain the correction according to the individual case needs.

### CBCT assessment

CBCT datasets were obtained using an i-CAT CBCT unit (Imaging Sciences International, Hatfield, PA). The source-to-detector distance was set at 67.5 cm. A voxel dimension of 0.3 mm was selected, utilizing a large field of view (17 cm) with settings of 120 kV, 18.54 mAs, and an exposure time of 8.9 s. The image detector was a flat panel measuring 20 × 25 cm, and images were captured at 14 bits during a single 360° rotation. Patients were instructed not to swallow or move during the scan. The raw images were exported into Digital Imaging and Communications in Medicine (DICOM) multifiles using i-CAT vision software. Subsequently, the DICOM files were reconstructed using Invivo software version 5.01 (Anatomage, San Jose, CA, USA).

CBCT images were acquired at two time points: prior to the insertion of the FFRD (T1) and 2 weeks following its removal (T2). The patient was instructed to bite in centric occlusion during imaging.

**Fig. 2:**
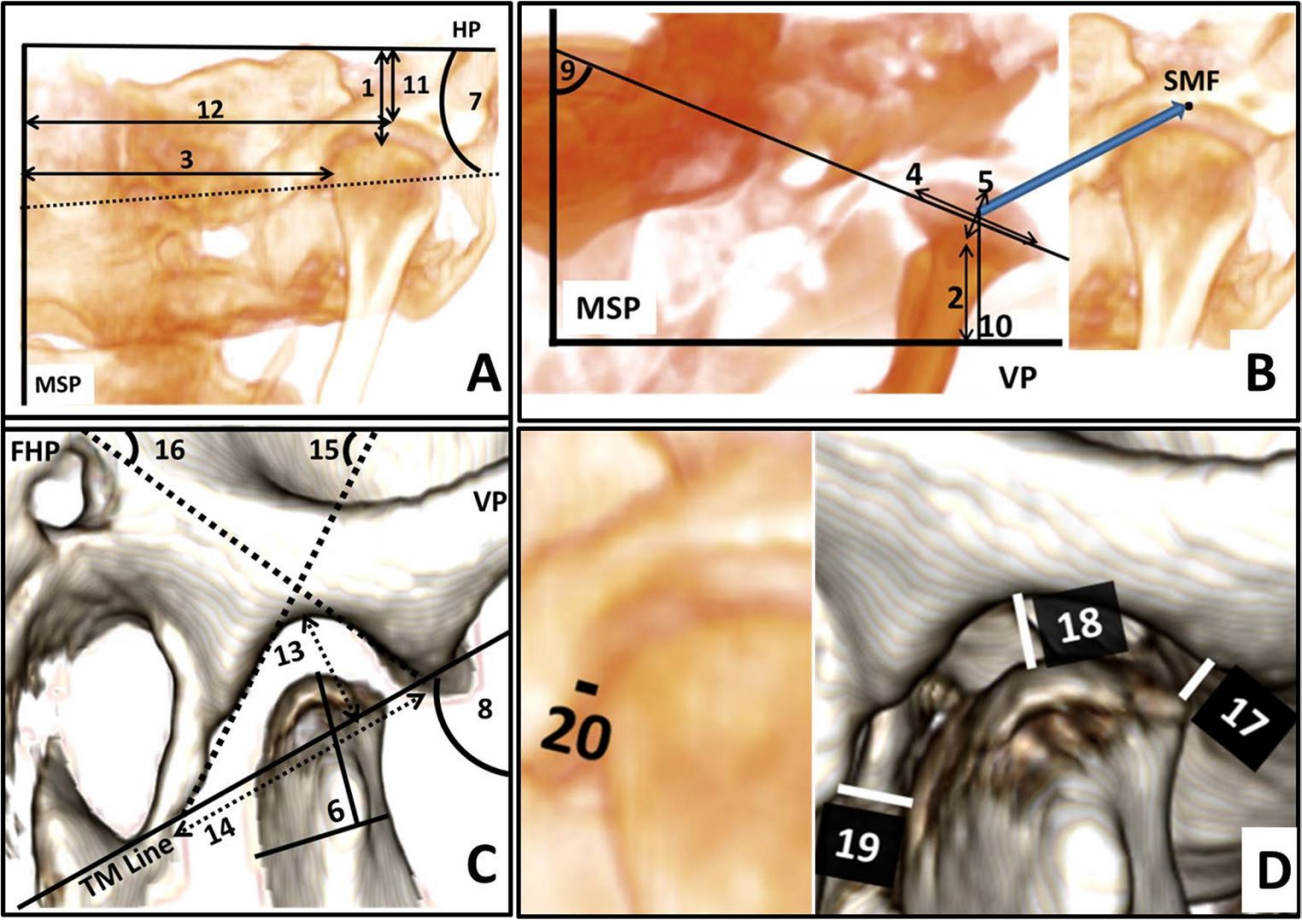
A: Coronal view measurements, B: Axial view measurements, C: Sagittal view measurements, and D: Combined views measurements showing: (1) V condylar position (mm), (2) AP condylar position (mm), (3) ML condylar position (mm), (4) Condylar length (mm), (5) Condylar width (mm), (6) Condylar height (mm), (7) ML Condylar inclination (°), (8) V Condylar inclination (°), (9) AP Condylar inclination (°), (10) AP MF position (mm). 11) V MF position (mm), 12) ML MF position (mm), 13) MF height (mm), 14) MF width (mm), 15) Anterior fossa line inclination (°), 16) Posterior fossa line inclination (°), 17) Anterior Joint Space (mm), 18) Superior Joint Space (mm), 19) Posterior Joint Space (mm), 20) Medial Joint Space (mm)

We customized a standardized analysis based on the TMJ analysis proposed in previous studies [35–38]. Landmarks were digitized in 3D volumetric images (Supplementary Material 1) and located in multiplanar projection; sagittal, coronal, and axial. Using these landmarks, we constructed reference planes and lines and identified three-dimensional measurements, which were registered on the 3D CBCT scan as detailed in Supplementary Material 2 and Fig. 2. An orthodontist and an oral and maxillofacial radiologist with over 10 years of experience (A.A.A.) conducted all linear and angular measurements in the 3D volumetric images. To assess intra- and inter-observer reliability, we reanalyzed the CBCT images 3 weeks after the initial measurement.

The primary outcome focused on changes in the mandibular condyle, specifically its dimensions, position, and inclination. Secondary outcomes encompassed: (a) changes in the GF, including dimensions, position, and wall inclinations, and (b) alterations in joint spaces, covering the anterior, superior, posterior, and medial joint spaces.

### Statistical analysis

Statistical analysis was conducted using SPSS Statistics Version 21 (Armonk, NY: IBM Corp.). To examine changes after the treatment and observation period within the same group, a paired samples t-test was employed. An independent samples t-test was used to compare changes between patient and control groups. Inter- and intra-observer reliability were assessed using Cronbach’s alpha reliability coefficient. The significance level for statistical tests was established at *P* < 0.05.

## Results

The treated group initially included 20 patients; however, three were lost to follow-up due to continuous appliance breakage. Consequently, 17 participants completed the treatment. The treatment with FFRD lasted, on average, 7.21 ± 1.3 months. For the control group, the interval between the two sets of images was 7.1 ± 3.2 months. The average pre-treatment ages were comparable between the treated group (14.2 ± 1.6 years) and the control group (13.5 ± 1.9 years). Intra- and inter-observer reliability demonstrated very good agreement, as detailed in Supplementary Material 3. Most TMJ data exhibited a parametric distribution, except for several parameters that followed a non-parametric distribution: mandibular fossa vertical position, condylar vertical position, condylar anteroposterior position, condylar length, condylar medial-lateral inclination, and all joint space measurements.

The baseline characteristics of both studied groups; age, CVMI stage, skeletal anteroposterior measurements, skeletal vertical measurements, and TMJ measurements are nearly comparable. However, the treated group exhibited a significantly higher medial joint space than the other group (Table 1).

**Table 1:** Comparison of the baseline TMJ measurements at T1 in the treated and control groups

Measurement		Treated group		Control group		*P*-value
		Mean	SD	Mean	SD	
**Age**		14.2	1.6	13.5	1.9	0.194
**Skeletal Measurements**						
**Growth stage**	CVMI	3.8	1.3	3.3	1.1	0.247
**Anteroposterior**	SNA (°)	79.51	2.84	82.42	2.05	0.055
	A-NV (mm)	3.01	4.49	3.62	2.12	0.959
	SNB (°)	72.64	3.11	75.68	1.88	0.054
	B-NV (mm)	−5.46	6.83	−3.68	1.98	0.480
	ANB (°)	6.79	1.45	6.75	1.23	0.929
	A-B Diff. (mm)	8.47	3.37	7.30	0.95	0.176
**Vertical**	Md/SN (°)	30.62	2.89	32.40	2.82	0.078
	MMP (°)	28.64	3.96	30.31	3.22	0.186
**Temporomandibular Measurements**						
**Condylar Position (mm)**	Vertical	1.01	3.93	2.59	1.31	**0.052**
	AP	−0.58	1.94	−1.38	1.61	**0.399**
	ML	49.65	2.38	47.74	2.93	**0.106**
**Condylar Inclination (°)**	ML	5.07	2.46	4.02	1.04	**0.387**
	Vertical	68.01	11.29	67.05	10.40	**0.834**
	AP	73.23	8.40	76.10	3.21	**0.338**
**Condylar dimension (mm)**	Length	15.58	2.73	15.88	1.96	**0.725**
	Width	7.06	1.29	7.23	0.91	**0.656**
	Height	8.21	1.10	8.38	1.80	**0.741**
**MF Position (mm)**	AP	−10.72	2.63	−9.30	1.35	**0.095**
	Vertical	0.58	1.27	1.53	0.60	**0.060**
	ML	46.70	2.00	45.45	2.00	**0.178**
**MF dimensions (mm)**	Height	8.14	1.52	7.75	0.76	**0.344**
	Width	19.57	10.14	18.80	1.66	**0.052**
**Mandibular FL inclination (°)**	AFL/FHP	56.23	7.28	56.91	10.44	**0.826**
	PFL/FHP	48.80	16.45	52.22	10.67	**0.477**
**Joint Spaces (JS) (mm)**	Anterior JS	1.48	2.55	2.14	0.52	**0.931**
	Superior JS	3.41	1.59	4.05	1.09	**0.414**
	Posterior JS	0.10	0.57	0.18	0.58	**0.517**
	Medial JS	0.23	4.27	3.24	0.91	0.006*

*: Significant atP ≤ 0.05

All comparisons of intervention and growth effects are detailed in Tables 2, 3 and 4. In the treated group, condylar length significantly increased from 15 ± 2.73 mm to 18.64 ± 9.79 mm (*P* = 0.031), whereas condylar width significantly decreased from 7.06 ± 1.29 mm to 6.54 ± 1.59 mm (*P* = 0.034). No other variables exhibited significant differences between T1 and T2 (Table 2). As depicted in Table 3, the control group experienced significant increases in both the mediolateral condylar position from 47.74 ± 2.93 mm to 48.28 ± 2.84 mm (*P* = 0.010) and the anterior wall inclination, which rose from 56.91 ± 10.44 degree to 59.86 ± 12.99 degree (*P* = 0.010).

**Table 2:** The TMJ measurements of the treated group before (T1) and after (T2) treatment

Measurement		T1		95% CI		T2		95% CI		*P*-value
		Mean	SD	Lower	Upper	Mean	SD	Lower	Upper	
Condylar Measurements										
Condylar Position (mm)	Vertical	1.01	3.93	−0.858	2.878	0.55	2.29	−0.539	1.639	**0.943**
	AP	−0.58	1.94	−1.502	0.342	−0.37	1.89	−1.268	0.528	**0.356**
	ML	49.65	2.38	48.519	50.781	51.16	4.28	49.125	53.195	**0.970**
Condylar Dimension (mm)	Length	15.58	2.73	13.620	17.539	18.64	9.79	13.986	23.294	0.031*
	Width	7.06	1.29	5.821	8.298	6.54	1.59	5.784	7.296	0.034*
	Height	8.21	1.10	6.886	9.533	8.03	1.09	7.512	8.548	**0.585**
Condylar Inclination (°)	ML	5.07	2.46	3.901	6.239	4.98	2.68	3.056	6.904	**0.733**
	Vertical	68.01	11.29	62.643	73.376	68.79	6.00	63.027	74.553	**0.767**
	AP	73.23	8.40	69.236	77.223	73.78	6.16	66.370	81.190	**0.809**
**Mandibular Fossa Measurements**										
MF Position (mm)	AP	−10.72	2.63	−14.522	−6.918	−10.50	2.38	−14.518	−6.482	**0.344**
	Vertical	0.58	1.27	−1.564	2.724	0.41	1.12	−1.752	2.572	**0.769**
	ML	46.70	2.00	42.839	50.561	45.93	1.92	41.758	50.102	**0.462**
MF dimension (mm)	Height	8.14	1.52	4.837	11.443	7.92	1.03	5.432	10.408	**0.382**
	Width	19.57	10.14	−4.925	44.065	16.76	2.81	9.290	24.230	**0.253**
MF lines Inclination (°)	AFL/TML	56.23	7.28	36.878	75.582	53.09	11.89	18.600	87.580	**0.248**
	PFL/TML	48.80	16.45	1.083	96.517	47.88	15.50	−0.841	96.601	**0.768**
**Joint Spaces Measurements**										
Joint Spaces (JS) (mm)	Anterior	−1.48	2.55	−6.535	9.495	−1.92	1.44	−2.605	−1.235	**0.877**
	Superior	1.41	1.59	−1.973	8.793	−0.53	2.51	−1.723	0.663	**0.02**
	Posterior	−0.10	0.57	−1.968	2.168	0.22	0.89	−0.203	0.643	**0.796**
	Medial	−0.23	4.27	−16.299	16.759	1.09	2.56	−0.127	2.307	**0.460**

*: Significant at P ≤ 0.05

**Table 3:** The TMJ measurements of the control group before (T1) and after (T2) observation period

Measurement		T1		95% CI		T2		95% CI		*P*-value
		Mean	SD	Lower	Upper	Mean	SD	Lower	Upper	
Condylar Measurements										
Condylar Position (mm)	Vertical	2.59	1.31	1.967	3.213	2.68	1.16	2.129	3.231	**0.460**
	AP	−1.38	1.61	−2.145	−0.615	−1.13	1.39	−1.791	−0.469	**0.227**
	ML	47.74	2.93	46.347	49.133	48.28	2.84	46.930	49.630	0.010*
Condylar Dimension (mm)	Length	15.88	1.96	14.472	17.287	16.11	1.90	15.207	17.013	**0.100**
	Width	7.23	0.91	6.355	8.104	7.40	0.88	6.982	7.818	**0.071**
	Height	8.38	1.80	6.214	10.545	8.24	1.59	7.484	8.996	**0.587**
Condylar Inclination (°)	ML	4.02	1.04	3.525	4.514	4.53	1.08	3.755	5.305	**0.401**
	Vertical	67.05	10.40	.62.106	71.993	70.82	5.02	65.999	75.641	**0.338**
	AP	76.10	3.21	74.574	77.6259	75.78	2.48	72.797	78.763	**0.591**
**Mandibular Fossa Measurements**										
MF Position (mm)	AP	−9.30	1.35	−11.251	−7.349	−9.33	1.62	−10.100	−8.560	**0.849**
	Vertical	1.53	0.60	0.517	2.543	1.68	0.58	1.404	1.956	**0.083**
	ML	45.45	2.00	41.589	49.311	45.36	1.81	44.500	46.220	**0.719**
MF dimension (mm)	Height	7.75	0.76	6.098	9.402	7.76	0.82	7.370	8.150	**0.880**
	Width	18.80	1.66	14.790	22.810	18.98	1.92	18.067	19.893	**0.230**
MF lines Inclination (°)	AFL/TML	56.91	10.44	29.158	84.662	59.86	12.99	53.685	66.035	0.010*
	PFL/TML	52.22	10.67	21.269	83.171	51.61	12.99	42.284	60.936	**0.741**
**Joint Spaces Measurements**										
Joint Spaces (JS) (mm)	Anterior	−2.14	0.52	0.506	3.774	−2.08	0.36	−2.513	−1.647	**0.605**
	Superior	4.05	1.09	0.359	7.741	4.38	1.27	2.544	6.216	**0.053**
	Posterior	−0.18	0.58	−1.924	2.284	−0.24	0.72	−1.455	0.975	**0.756**
	Medial	3.24	0.91	−0.282	6.762	3.38	1.06	1.334	5.426	**0.233**

*: Significant at P ≤ 0.05

**Table 4:** Comparison of the TMJ measurements changes of the treated and control groups

Measurement		Treatment				Control				Treatment VS Control
		Diff (T2-T1)		95% CI		Diff (T2-T1)		95% CI		*P*-value
		Mean	SD	Lower	Upper	Mean	SD	Lower	Upper	
**Condylar Measurements**										
Condylar Position (mm)	Vertical	−0.46	4.38	−2.542	1.622	0.09	0.55	−0.838	1.018	**0.718**
	AP	0.22	1.01	−0.260	0.700	0.24	0.73	−1.169	1.649	**0.836**
	ML	0.02	0.88	−0.398	0.438	0.54	0.77	−1.133	2.213	**0.266**
Condylar Dimension (mm)	Length	0.67	1.71	−0.143	1.483	0.23	0.50	−0.978	1.438	**0.610**
	Width	−0.52	0.92	−0.957	−0.083	0.17	0.35	−0.760	1.100	0.001*
	Height	−0.18	1.31	−0.803	0.443	−0.14	1.06	−3.215	2.935	**0.570**
Condylar Inclination (°)	ML	−0.08	2.54	−1.903	1.743	0.51	1.71	−4.865	5.885	**0.297**
	Vertical	0.77	10.58	−9.391	10.931	3.76	11.08	−33.755	41.275	**0.609**
	AP	0.55	9.13	−10.433	11.533	−0.31	1.67	−6.369	5.749	**0.483**
**Mandibular Fossa Measurements**										
MF Position (mm)	AP	0.22	0.93	−0.222	0.662	−0.03	0.69	−0.860	0.800	**0.718**
	Vertical	−0.17	1.49	−0.878	0.538	0.15	0.29	−0.269	0.569	**0.469**
	ML	0.43	1.03	−0.060	0.920	−0.09	0.98	−1.744	1.564	**0.179**
MF dimension (mm)	Height	−0.22	1.03	−0.710	0.270	0.01	0.28	−0.531	0.551	**0.642**
	Width	−2.81	9.77	−7.454	1.834	0.18	0.58	−1.080	1.440	**0.174**
MF lines Inclination (°)	AFL/TML	−3.13	10.77	−8.250	1.990	2.95	4.14	−7.051	12.951	0.003*
	PFL/TML	−0.91	12.56	−9.927	8.107	−0.61	7.53	−20.626	19.406	**0.5936**
**Joint Spaces Measurements**										
Joint Spaces (JS) (mm)	Anterior	−0.44	2.92	−1.828	0.948	0.06	0.56	−0.206	0.326	**0.564**
	Superior	1.94	3.26	0.390	3.490	0.33	0.52	−0.043	0.703	**0.32**
	Posterior	0.31	1.11	−0.218	0.838	−0.06	0.56	−0.598	0.478	**0.746**
	Medial	1.32	4.62	−0.876	3.516	0.14	0.56	−0.534	0.814	**0.623**

*: Significant at P ≤ 0.05

In comparing the mean change between the treated and control groups (Table 4), the condylar width decreased significantly in the treatment group by 0.52 ± 0.92 mm, compared to an increase of 0.17 ± 0.35 mm in the control group (*P* = 0.010). Furthermore, the anterior wall inclination also decreased significantly in the treatment group by 3.13 ± 10.77 degree, in contrast to an increase of 2.95 ± 4.1 degree in the control group (*P* = 0.003). All other measurements showed no significant differences between the two groups.

## Discussion

This study aimed to offer a 3D evaluation of the positional and dimensional changes in the osseous components of the TMJ influenced by the FFRD. A previous study [33] addressed this objective, but it was limited methodologically by focusing solely on joint space measurements using CT scans. Additionally, the image analysis relied on estimating joint space area with the Cavalieri principle [39]. These estimates were then input into a computer program to determine the volumes of the anatomical structures measured.

The current study specifically included female participants. This choice can be justified by the distinct patterns of mandibular growth observed in males and females [40]. A recently published systematic review highlights the necessity of reporting the effects of gender on the response to treatment with functional appliances separately for each gender [41].

Concerning the condylar outcomes, the results indicated changes, some of which were statistically significant but clinically insignificant. All changes measured less than 1 mm and, therefore, had no impact on treatment outcomes, particularly regarding the potential development of TMDs. This finding supports studies indicating that FFRD treatment does not contribute to the risk of TMJ dysfunction in patients without pre-existing signs or symptoms [42]. However, a pilot study by LeCornu et al. [43], involving seven patients treated with the Herbst appliance for an average of 13 months, reported anterior displacement of the condyle ranging from 2.5 to 2.9 mm. Additionally, Varghese et al. concluded that both the FFRD and powerscope appliances are effective and cost-efficient for treating Class II skeletal malocclusion, with anterior mandibular displacement accounting for 3.16 mm out of a total of 4.90 mm achieved using the FFRD [44].

Similar findings were reported in a retrospective study evaluating the Sander Bite Jumping Appliance, which had a treatment duration of 1.6 ± 0.6 years [45]. The study found significant superior and posterior growth in the condylar region, with considerable individual variability. This variability might be attributed to the longer treatment duration compared to the current study, suggesting that treatment exceeding 6 months may enhance skeletal changes in the condyles.

Shalu et al. estimated that the improvement in articular position for Class II cases treated with FFRD was 1.8 mm, although this result was not statistically significant. The opening of the articular angle contributes to forward mandibular movement, reducing overjet and enabling molar correction [1]. Additionally, they found that the change in condylar length with FFRD had a mean difference of 1.08 mm. Though this change was not statistically significant, it may relate to the altered mandibular position induced by fixed functional therapy via the relocation of the articular point [1]. The forward positioning of the mandible could similarly result from the relocation of the articular point at the condylar region in both appliances [1]. Furthermore, Kumawat et al. showed that FFRD caused a more anterior and downward shift in the mandibular condylar position compared to the power scope group, although this difference was not statistically significant between the two appliances [46].

The comparisons of mandibular fossa width and height revealed no significant differences, both within and between the groups, indicating that neither the FFA nor growth had an effect. This finding aligns with the study by Kinzinger et al., which evaluated the impact of treatment using a functional mandibular advancer on mandibular fossa morphology through magnetic resonance imaging (MRI) [47]. They concluded that treatment did not significantly alter either the width (*P* = 0.804) or the depth (*P* = 0.286) of the mandibular fossa.

The only noteworthy outcome in the MF changes pertains to the inclination of the anterior wall. In the control group, this inclination increased, indicating posterior remodeling of the MF, as noted during the evaluation of Class II subjects’ growth patterns [48]. Conversely, the treated group displayed a statistically insignificant decrease, suggesting anterior remodeling of the MF’s anterior wall. A significant difference emerged when comparing this outcome between the treated and control groups. Kinzinger et al. did not assess the inclination of the mandibular fossa walls, focusing solely on metric measurements [47]. They reported no significant changes in the anterior (*P* = 0.804) or posterior (*P* = 0.605) wall dimensions. This suggests that the FFA may have prevented posterior remodeling of the MF’s anterior wall, thereby contributing to the correction of Class II malocclusion. This finding aligns with the hypothesized effect of the Herbst appliance, which is thought to involve anterior fossa and/or posterior condylar remodeling [30]. LeCornu et al. [43] observed bone resorption and deposition at the anterior (1.4–1.7 mm) and posterior (0.6–0.8 mm) surfaces of the GF with Herbst treatment.

The varying durations of treatment between the control group and the current study may account for the discrepancies in findings. Aras et al. [49] reported a significant change in articular disc position and noted a tendency for the disc to progressively reposition in relation to the condyle. Their MRI results indicated that the condyle-fossa relationship remains stable following FFRD treatment, possibly due to appositional growth at the condyle and glenoid fossa. In contrast, Parvathy et al. [31] found no clear evidence of remodeling at the condyle and glenoid fossa. Additionally, Insabralde et al. reported that the Herbst appliance repositions the jaw in a more anterior and downward direction, moving the condyles away from the articular eminence. This repositioning is believed to facilitate condylar bone apposition, thereby increasing the overall dimensions of mandibular size [5].

The impact on TMJ function, whether it causes, worsens, or treats TMJ disorders– holds more significance than merely morphological changes. Although such changes can contribute to the development of TMJ disorders, evidence suggests that positioning the mandible more forward than the intercuspal position can effectively address certain disc derangement issues. This forward displacement of the condyle helps restore an optimal condyle-disc relationship, facilitating better tissue adaptation and repair [50]. Isola et al. reported significant improvements in various TMJ signs and symptoms following the use of an orthodontic functional appliance over 24 months in patients with juvenile idiopathic arthritis (JIA) and TMJ disorders [51].

Regarding joint spaces measurements, none of the assessed outcomes were significant. Kinzinger et al. [52] found no changes in both anterior and posterior joint spaces with FFA treatment. In contrast, Parvathy et al. [31] observed that FFRD led to changes in TMJ structures, noting an increase in condylar height and width, accompanied by a decrease in anterior and superior joint spaces. However, when comparing it with the twin block appliance, they determined that the twin block is more efficient. Arici et al. [33] reported that anterior joint space volume increased by 38% in the treated group and 20% in the control group, while posterior JS volume decreased by 9% in the treated group and increased by 2% in the control group. It is important to note that the volumetric approach used has not been validated and yields conflicting evidence when compared to more accepted methods [18].

In our study, negative changes in the anterior wall inclination of the mandibular fossa suggest a geometric predisposition to TMDs by decreasing the slope of the anterior wall, making the disc more susceptible to anterior displacement, especially when accompanied with the reported changes in the condylar width and medial joint spaces. An MRI assessment is necessary to confirm the possible effect and correlate with the clinical assessment of the TMJ.

It’s important to emphasize that the osseous changes at the TMJ level are not the sole factors influencing the extent of potential correction for skeletal malocclusion, the likelihood of relapse, and how treatment impacts cases predisposed to TMDs. Kiliaridis et al. [53] evaluated masseter muscle thickness as a predictive factor for treatment outcomes with the twin-block functional appliance and observed changes in masseter thickness during treatment. Their findings indicated that weaker muscles lead to greater dentoalveolar effects, and vice versa. Moreover, functional appliances tend to cause thinning of these muscles in the short term after treatment.

Another critical aspect often overlooked in orthodontic diagnosis and treatment planning is the evaluation of the centric relation-maximum intercuspation (CR-MI) discrepancy. Lim et al. [54] found that all patients with significant CR-MI discrepancies exhibited TMJ disk displacement. Assessing this factor before treatment and correlating it with existing findings necessitates a more cautious approach when using FFA. If the decision is made to proceed with FFA, it is essential to ensure the stable musculoskeletal position of the condyles in the fossa, focusing on accurate orthodontic diagnosis and the prevention of TMD development [55, 56].

In summary, it is essential to evaluate all the aforementioned factors and consider them as qualifying criteria for cases selected for functional appliance therapy. These criteria include: the absence of tight ligaments, adequate thickness of the masseters and supplementary muscles, a stable musculoskeletal position of the condyles in the glenoid fossa, a favorable dimensional interrelationship between the joint structures, favorable glenoid fossa morphology (Class II division 2 cases might be excluded due to unfavourable morphology), and the absence of gross morphological symmetries between the right and left sides under the same applied forces. These factors can be initially set as selection criteria and evaluated clinically by high-quality clinical evidence for evidence-based supported research.

Although the sample size was calculated in advance, it remains a limitation of this study. We recommend conducting studies with larger sample sizes that analyze predictive factors. The current study assessed the short-term effect of the FFRD on the TMJ; however, long-term studies might yield more robust findings. Methodological limitations also include the impossibility of applying blinding during the intervention. Furthermore, the generalizability of the findings is limited because the sample consisted only of female patients, whereas males might respond differently. This study did not assess the intervention’s effect on TMDs, as such cases were excluded. Future research should consider including TMD cases and evaluating the status of TMDs following fixed functional treatment. A long-term assessment of muscular changes, including muscle dimensions and functional activity, along with an evaluation of osseous changes, backed by thorough clinical examinations, is recommended to provide a complete understanding of the effects and side effects of fixed functional appliances. These outcomes are to be evaluated on each side because identical TMJ morphology as well as an identical response to treatment is almost impossible.

## Conclusions

A short-term clinical trial demonstrated that functional stimulation with the FFRD did not result in significant TMJ remodeling changes. However, extending the follow-up period beyond 6 months could produce different outcomes, necessitating further research.

## Supplementary Information

Below is the link to the electronic supplementary material.

ESM 1 (DOCX 29.4 KB)

ESM 2 (DOCX 30.0 KB)

ESM 3 (DOCX 30.5 KB)

## Data Availability

No datasets were generated or analysed during the current study.
