# Supplementary material for: Positional and dimensional temporomandibular joint osseous changes in patients treated with the forsus fatigue resistant device: a non-randomized clinical trial
Source: Clin Oral Investig. 2025 Aug 18;29(9):414. doi: 10.1007/s00784-025-06474-3 (PMC12358331; doi:10.1007/s00784-025-06474-3)
Supplement: Supplementary file 1 — (DOCX 29.4 KB) [file 784_2025_6474_MOESM1_ESM.docx]

Supplementary table 1: Description of landmarks identified in the Cone Beam Computed Tomography Analysis

| **Landmark (Abbreviation)** | **Definition** |
| --- | --- |
| **Craniofacial Landmarks** | |
| Orbitale (Or) (Right and Left) | The most inferior point of each infra-orbital rim |
| Porion (Po) (Right and Left) | The most outer superior bony points of the EAM |
| Sella (S) | Center point of the pituitary fossa |
| Nasion (Na) | The most anterior midpoint of the fronto-nasal suture. |
| Subspinale (A) | The deepest point on the upper jaw is between the supradentale and the anterior nasal spine |
| Submenale (B) | The lowest point between the infradentale and the pogonion on the lower jaw curvature |
| Gonion (Go) | The posterior and inferior borders of the mandible are bisected at each angle to ascertain the midpoint of the gonion angle |
| Menton (Me) | The mandibular symphysis’s most inferior point |
| Anterior nasal spine (ANS) | The most anterior point of the anterior nasal spine of the maxilla. |
| Posterior nasal spine (PNS) | The most posterior point of the posterior nasal spine of the palatine bone. |
| **Condylar Landmarks (CP)** | |
| Superior CP (SCP) | The most superior midpoint of the condylar head. |
| Lateral CP (LCP) | The most lateral point on the condylar head. |
| Medial CP (MCP) | The most medial point on the condylar head. |
| Anterior CP (ACP) | The most anterior point on the condyle. |
| Posterior CP (PCP) | The most posterior point on the condyle. |
| Geometric Center (GC) | Center of the condyle. |
| Anterior Neck Point (ANP) | The deepest anterior point of the condylar neck. |
| Posterior Neck Point (PNP) | The most posterior point of the condylar neck. |
| CW anterior point (CWa) | The most anterior point of the condyle at its maximum width |
| CW posterior point (CWp) | The most posterior point of the condyle at its maximum width |
| **Mandibular Fossa (MF) Landmarks** | |
| Superior MF point (SMF) | The most superior and midpoint of the bony MF. |
| Anterior Tubercle (AT) | Most inferior and posterior point on articular tubercle. |
| Inferior Meatus (IM) | The most inferior and lateral point on the EAM. |
| AF Point (AFP) | The most anterior inferior point in the anterior wall of the MF. |
| PF Point (PFP) | The most posterior inferior point in the posterior wall of the MF. |
| Superior point of Anterior Fossa inclination (AFIs) | The most superior point on a line tangent to the most posterior MF area, opposite to the anterior condylar area. |
| Inferior point of Anterior fossa inclination (AFIi) | The most inferior point on a line tangent to the most posterior MF area, opposite to the anterior condylar area. |
| Superior point of Posterior fossa inclination (AFIs) | The most superior point on a line tangent to the most anterior MF area, opposite to the posterior condylar area. |
| Inferior point of Posterior fossa inclination (PFIi) | The most inferior point on a line tangent to the most anterior MF area, opposite to the posterior condylar area. |
| **Joint Spaces Landmarks** | |
| AJS Fossa Point (AJSf) | The most posterior point of the anterior wall of the MF * |
| AJS condylar point (AJSc) | The most anterior point of the condyle * |
| PJS fossa point (PJSf) | The most anterior point of the posterior wall of the MF* |
| PJS condylar point (PJSc) | The most posterior point of the condyle * |
| MJS fossa point (MJSf) | The most lateral point of the medial wall of MF.* |

AF= Anterior Fossa, AJS= Anterior Joint space, CW= Condylar Width, CP= Condylar Point, MF= Mandibular Fossa, MJS= Medial Joint Space, PF= Posterior Fossa, PJS= Posterior Joint Space.

* Opposed to the shortest anterior condylar-fossa distance.
