# Supplementary material for: Positional and dimensional temporomandibular joint osseous changes in patients treated with the forsus fatigue resistant device: a non-randomized clinical trial
Source: Clin Oral Investig. 2025 Aug 18;29(9):414. doi: 10.1007/s00784-025-06474-3 (PMC12358331; doi:10.1007/s00784-025-06474-3)
Supplement: Supplementary file 2 — (DOCX 30.0 KB) [file 784_2025_6474_MOESM2_ESM.docx]

Supplementary table 2: Description of Planes/Lines constructed and measurements used in the Cone Beam Computed Tomography Analysis

| **Plane/Line/ Measurement (Abbreviation)** | **Definition** |
| --- | --- |
| Horizontal plane (HP) | Plane defined by 3 landmarks: right Or, left, and right Po |
| Midsagittal plane (MSP) | Plane through S and Nasion, perpendicular to the HP |
| Vertical plane (VP) | Plane through S; perpendicular to the HP and MSP |
| Anterior cranial base (S-N) | Distance is measured between points S and N |
| N vertical plane (NV) | The perpendicular plane from the nasion point through the Frankfort horizontal |
| Palatal plane (PP) | Plane from the anterior to the posterior nasal spine |
| Mandibular plane (MP) | Plane from Gonion to Gnathion landmarks |
| MF horizontal plane (MFP) | Plane through SMF point; parallel to the HP |
| Anterior fossa line (AFL) | Line from AFLs to AFLi |
| Posterior fossa line (PFL) | Line from PFLs to PFLi |
| Tuberculo-meatal line (TML) | Line from AT to IM. |
| AP condylar line (ACP-PCP) | Line from ACP to PCP |
| ML condylar line (MCP-LCP) | Line from MCP to LCP |
| Condylar neckline (ACN-PCN) | Line from ACN to PCN |
| **Skeletal Measurements** | |
| SNA (°) | The angle formed between the NA line and the SN plane |
| A-NV (mm) | Distance is measured from point A to the nasion-perpendicular line to the FH plane |
| SNB (°) | The angle formed between the NB line and the SN plane |
| B-NV (mm) | Distance is measured from point B to the nasion-perpendicular line to the FH plane |
| ANB (°) | The angle formed by the intersection of the NA and NB lines |
| A-B Diff. (mm) | The difference between the A-NV and B-NV measurements |
| Md/SN (°) | The angle formed between GoGn and SN lines |
| MMP (°) | The angle formed between the palatal and mandibular plans |
| **Condylar Measurements** | |
| V condylar position (mm) | Vertical distance between the SCP and HP |
| AP condylar position (mm) | AP distance between the ACP and VP |
| ML condylar position (mm) | The ML distance between the Cd_c_ and MSP |
| Condylar length (mm) | Distance between MCP and LCP |
| Condylar width (mm) | Distance between CWa and CWp |
| Condylar height (mm) | Perpendicular distance between SCP and ACN-PCN |
| ML Condylar inclination (°) | Angle between MCP-LCP and HP |
| V Condylar inclination (°) | Angle between ACP-PCP and VP |
| AP Condylar inclination (°) | Angle between MCP-LCP and MSP |
| **Mandibular Fossa Measurements** | |
| AP MF position (mm) | Perpendicular distance between SMF and VP |
| V MF position (mm) | Perpendicular distance between SMF and HP |
| ML MF position (mm) | Perpendicular distance between SMF and MSP |
| MF height (mm) | Perpendicular distance between SMF and TM line |
| MF width (mm) | Distance between AFP and PFP |
| Anterior fossa line inclination (°) | Anterior angle between AFL and TM line |
| Posterior fossa line inclination (°) | Anterior angle between PFL and TM line |
| **Joint Space Measurements** | |
| Anterior Joint Space (mm) | shortest distance between AJSf and AJSc |
| Superior Joint Space (mm) | shortest distance between MF and SCP |
| Posterior Joint Space (mm) | shortest distance between PJSf and PJSc |
| Medial Joint Space (mm) | shortest distance between MJSf and MCP |

AP = Anteroposterior, ML= Medio-lateral, V= Vertical
