# Supplementary material for: Positional and dimensional temporomandibular joint osseous changes in patients treated with the forsus fatigue resistant device: a non-randomized clinical trial
Source: Clin Oral Investig. 2025 Aug 18;29(9):414. doi: 10.1007/s00784-025-06474-3 (PMC12358331; doi:10.1007/s00784-025-06474-3)
Supplement: Supplementary file 3 — (DOCX 30.5 KB) [file 784_2025_6474_MOESM3_ESM.docx]

Supplementary material 3: Results of Cronbach’s alpha reliability coefficient for intra- and inter-observer agreement (reliability) for skeletal and TMJ measurements

| **Category** | **Measurement** | **Intra-observer** | **Inter-observer** |
| --- | --- | --- | --- |
| **Skeletal** | SNA (°) | 0.995 | 0.9**85** |
|  | A-NV (mm) | 0.998 | 0.997 |
|  | SNB (°) | 0.999 | 0.995 |
|  | B-NV (mm) | 0.869 | 0.998 |
|  | ANB (°) | 0.998 | 0.978 |
|  | A-B Diff. (mm) | 0.995 | 0.999 |
|  | Md/SN (°) | 0.995 | 0.9**85** |
|  | MMP (°) | 0.998 | 0.997 |
| **Condylar Position (mm)** | Vertical | 0.792 | 0.788 |
|  | AP | 0.839 | 0.824 |
|  | ML | 0.817 | 0.800 |
| **Condylar Inclination (°)** | ML | 0.869 | 0.824 |
|  | Vertical | 0.877 | 0.859 |
|  | AP | 0.880 | 0.871 |
| **Condylar Dimension (mm)** | Length | 0.829 | 0.824 |
|  | Width | 0.869 | 0.842 |
|  | Height | 0.790 | 0.782 |
| **Mandibular Fossa Position (mm)** | AP | 0.833 | 0.830 |
|  | Vertical | 0.789 | 0.771 |
|  | ML | 0.819 | 0.810 |
| **Mandibular Fossa dimensions (mm)** | Height | 0.886 | 0.814 |
|  | Width | 0.792 | 0.788 |
| **Mandibular Fossa inclination (°)** | AFL/FHP | 0.800 | 0.782 |
|  | PFL/FHP | 0.816 | 0.807 |
| **Joint Spaces (JS) (mm)** | Anterior JS | 0.853 | 0.845 |
|  | Superior JS | 0.841 | 0.829 |
|  | Posterior JS | 0.830 | 0.825 |
|  | Medial JS | 0.847 | 0.841 |
